# New moss taxa from Eastern Himalaya: Plastid genomics supports the establishment of Scabridentaceae fam. nov. and the description of *Scabridens gaochienii* sp. nov. (Hypnales, Bryophyta)

**DOI:** 10.3389/fpls.2026.1842480

**Published:** 2026-06-09

**Authors:** Wen-Zhuan Huang, Yu-Si Liu, Xin-Yin Ma, Qing-Hua Wang, Wen-Zhang Ma, Rui-Liang Zhu, Tian-Xiong Zheng, Yu-Huan Wu

**Affiliations:** 1 College of Life Sciences, South China Agricultural University, Guangzhou, China; 2 College of Life and Environmental Sciences, Hangzhou Normal University, Hangzhou, China; 3 Zhejiang Provincial Key Laboratory of Wetland Intelligent Monitoring and Ecological Restoration, Hangzhou Normal University, Hangzhou, China; 4 State Key Laboratory of Plant Diversity and Specialty Crops, Institute of Botany, Chinese Academy of Sciences, Beijing, China; 5 Herbarium, Key Laboratory for Plant Diversity and Biogeography of East Asia, Kunming Institute of Botany, Chinese Academy of Sciences, Kunming, Yunnan, China; 6 Bryology Laboratory, Department of Biology, School of Life Sciences, East China Normal University, Shanghai, China; 7 Hattori Botanical Laboratory, Nichinan, Japan

**Keywords:** China, chloroplast genome, mosses, new family, new species

## Abstract

The Himalayas represent a globally significant biodiversity hotspot, yet bryophyte diversity remains insufficiently explored, especially in some inaccessible areas. During recent field surveys in Motuo County, Xizang, China, we collected an unusual moss species resembling *Scabridens* E.B.Bartram but differing in gametophyte morphology, most notably by a short double costa. To clarify its taxonomic position, we assembled and annotated 20 complete chloroplast genomes from Cryphaeaceae, Leucodontaceae, and Pterobryaceae, and analyzed these alongside 29 additional Hypnales accessions. Comparative plastome analyses revealed conserved structures across these lineages, with minor variations in genome size, GC content, and IR boundary regions. Phylogenomic analyses revealed the unknown taxon as sister to *S*. *sinensis* E.B.Bartram with strong support. Morphologically, it differs from *S*. *sinensis* by a short double costa, leaf margins revolute from the base to near the apex, and longer setae. Both morphological and molecular evidence support the recognition of this unknown taxon as a new species, which we described here as *S*. *gaochienii* W.Z.Huang & Y.Huan Wu. Furthermore, although *Scabridens* is sister to Cryphaeaceae in plastid phylogenies, its pronounced morphological distinctiveness supports the recognition of a new family, Scabridentaceae W.Z.Huang & Y.Huan Wu, to accommodate the genus. Our results highlight the power of plastid phylogenomics in resolving complex relationships in Hypnales and underscore the eastern Himalayas as a priority region for bryophyte diversity exploration.

## 1 Introduction

The Himalayas are recognized as one of the world’s major biodiversity hotspots (Mittermeier et al., 2004; Orme et al., 2005) and rank among the most bryophyte-rich regions globally (Groombridge, 1992). Characterized by lush vegetation, this region serves as a repository for numerous globally significant plant species (Kandel et al., 2019), and has long been known as the “world’s biological gene bank” (Zhu et al., 2023). Motuo County, located in the eastern Himalayas, encompasses an exceptional elevation gradient of 7,000 m, ranging from tropical rainforests to a permanent glacier, and supports highly heterogeneous habitats with abundant light, heat, and water resources. This county was the last area to be connected to the outside by highway, with the road completed in 2013. Due to its difficult access, biodiversity surveys have been limited, and many areas remain poorly explored. In recent years, several new taxa have been described from this region based on recent collections, such as *Vaccinium motuoense* Y.H.Tong & Y.J.Guo (Tong et al., 2021), *Ficus motuoensis* Zhen Zhang & Hong Qing Li (Zhang et al., 2022), *Stellaria motuoensis* Meng Li & Y.F.Song (Li et al., 2022), *Kuhlhasseltia motuoensis* X.H.Jin & C.Ye (Ye et al., 2023), *Uncifera motuoensis* M.Z.Huang, Xue D.Chen & M.K.Li (Huang et al., 2024), etc. However, few of these discoveries pertain to bryophytes, with the exception of *Dicranum motuoense* W.Z.Huang, Tubanova & Y.Huan Wu (Huang et al., 2025), *Pseudotrachypus motuoensis* Y.X.Tian, Q.Zuo & Li Zhang (Tian et al., 2025), and *Ptychanthus striatus* var. *motuoensis* Jian Wang bis, Gradst. & R.L.Zhu (Dai et al., 2021). Obviously, the bryophyte diversity of Motuo County remains substantially underestimated.

Leucodontaceae is a family of mosses primarily distributed in the temperate zones, often growing on tree trunks and branches (Zhang, 2011a). Currently, this family comprises five genera worldwide (Brinda and Atwood, 2026): three monotypic genera (*Dozya* Sande Lac., *Eoleucodon* H.A.Mill. & H.Whittier, and *Scabridens* E.B.Bartram); *Pterogoniadelphus* M.Fleisch. with two species; and *Leucodon* Schwägr. with 33 species. *Scabridens* is a genus endemic to China, consisting solely of one rare species, *S. sinensis* E.B.Bartram (Bartram, 1935), found in the southwestern region (Zhang, 2011a; Jia and He, 2013). Morphologically, the genus is characterized by erect and simple stems, a single long costa, papillose peristome teeth, smooth capsules, serrate upper leaf margins, and an autoicous sexual condition (Bartram, 1935; Frey and Stech, 2009). Notably, this sexual condition is unusual in Leucodontaceae, where all other taxa are dioicous. Recent plastome-based phylogenetic analyses have shown that *Scabridens* and *Leucodon* Schwägr. do not form a monophyletic group; instead, *Scabridens* is revealed as sister to Cryphaeaceae (Li et al., 2024). However, as the study did not discuss this topological relationship or propose corresponding taxonomic treatments, the systematic placement of *Scabridens* remains unresolved and requires further reassessment.

During our fieldwork in Motuo County, Xizang, China (Huang et al., 2025; Tian et al., 2025), we collected an unusual moss with gametophytic features reminiscent of *Leucodon*, including clearly differentiated alar cells and a short double costa (Frey and Stech, 2009; Zhang, 2011a). However, its sporophytic features are markedly different, such as a reduced endostome, papillose exostome teeth, and an autoicous sexual condition, which are more consistent with *Scabridens* (Bartram, 1935; Frey and Stech, 2009; Zhang, 2011a). Nevertheless, this unknown taxon can be distinguished from known *Scabridens* species by a unique combination of traits, notably a short double costa and leaf margins strongly revolute from the base to near the apex. These unique morphological characters prompted further integrative investigation.

The reconstruction of a reliable and unequivocal phylogeny of bryophytes is essential for elucidating the mechanisms underlying plant terrestrialization (Bechteler et al., 2023; Shen et al., 2024; Xu et al., 2026). However, phylogenetic reconstructions based on limited DNA loci are inadequate to resolve the relationships within Hypnales (Cox et al., 2010; Li et al., 2024). Compared with short DNA markers, complete chloroplast genomes provide substantially more phylogenetically informative characters and greater structural stability, and have emerged as powerful tools for resolving bryophyte evolution relationships (Yu et al., 2019; Xiang et al., 2022; Li et al., 2024; Xu et al., 2026). In this study, we conduct a plastid phylogenomic investigation of Hypnales, with a particular focus on the Cryphaeaceae, Leucodontaceae, and Pterobryaceae clades, by integrating newly sequenced and publicly available plastome data across 18 families. Specifically, we aim to: (1) generate and annotate complete plastomes for 20 newly sampled accessions within the focal clades; (2) characterize plastome evolution in these clades through cpmparative analyses of genome structure and sequence variation; (3) test the taxonomic status of the Motuo taxon, and evaluate whether it represents a new species using combined morphological and phylogenetic evidence; and (4) reassess higher-level relationships and family delimitation surrounding *Scabridens*, and provide formal taxonomic revisions where warranted.

## 2 Material and methods

### 2.1 Taxon sampling and sequencing

A total of 20 specimens were newly collected and sequenced for this study, including eight from Cryphaeaceae, five from Leucodontaceae, two from Pterobryaceae, four representing *Scabridens*, and one unidentified lineage from the Himalayas (**Table 1**). All specimens were identified by Wen-Zhuan Huang. Voucher specimens are deposited in the Herbarium of Hangzhou Normal University (HTC), the Herbarium of East China Normal University (HSNU), and the Herbarium, Institute of Botany, CAS (PE). To broaden the phylogenetic context, 29 additional Hypnales accessions were included, and three Orthotrichaceae accessions were selected as outgroups.

**Table 1:** Taxa, DNA code, voucher, herbarium acronyms, localities, and GenBank accession numbers of species newly sequenced in this study.

No.	Species	DNA Code	Vouchers (herbarium code)	Locality	GenBank
1.	*Cyptodontopsis leveillei*	HTC478	*W.-Z. Ma 13-5136* (KUN)	China: Yunnan	PZ196757
2.	*Dozya japonica*	HTC422	*T. Yamaguchi 42638* (HIRO)	Japan: Kyushu	PZ196758
3.	*Leucodon exaltatus*	HTC421	*W.-Z. Huang 20250928-15* (HTC)	China: Xizang.	PZ196759
4.	*Leucodon sciuroides*	HTC305	*M. Sulayman 281783* (XJU)	China: Xinjiang	PZ196760
5.	*Leucodon secundus* var. *strictus*	HTC318	*W.-Z. Huang 20250830-29* (HTC)	China: Qinghai	PZ196761
6.	*Leucodon secundus* var. *strictus*	HTC393	*W.-Z. Huang 20250929-110* (HTC)	China: Xizang	PZ196762
7.	*Pilotrichopsis dentata*	HTC151	*W.-Z. Haung et al., 20250322-18* (HTC)	China: Zhejiang	PZ196763
8.	*Pterobryopsis acuminata*	HTC419	*W.-Z. Huang 20250929-115* (HTC)	China: Xizang	PZ196764
9.	*Scabridens sinensis*	HTC166	*W.-Z. Ma 20-10936* (KUN)	China: Xizang	PZ196765
10.	*Scabridens sinensis*	HTC167	*W.-Z. Ma 19-10918* (KUN)	China: Xizang	PZ196766
11.	*Scabridens sinensis*	C89	*L. Lian 206* (PE)	China: Sichuan	PZ196767
12.	*Scabridens sinensis*	HTC168	*W.-Z. Ma 15253* (KUN)	China: Sichuan	PZ196768
13.	*Scabridens gaochienii*	HTC083	*W.-Z. Huang & F.-Y. Zhang 20241012-71* (HTC)	China: Xizang	PZ196769
14.	*Schoenobryum concavifolium*	H53	*W.-Z. Huang & S.-H. Lu 20210908-26* (HSNU)	China: Yunnan	PZ196770
15.	*Schoenobryum concavifolium*	HTC488	*W.-Z. Ma 12588* (KUN)	China: Yunnan	PZ196771
16.	*Schoenobryum concavifolium*	HTC489	*J.R. Shevock 54140* (KUN)	China: Yunnan	PZ196772
17.	*Schoenobryum concavifolium*	W16	*Q.-H. Wang 2002* (PE)	China: Yunnan	PZ196773
18.	*Sphaerotheciella sphaerocarpa*	C88	*M.-Z. Wang 202104-0792* (PE)	China: Sichuan	PZ196774
19.	*Sphaerotheciella sinensis*	H50	*W.-Z. Huang et al., 20210529-19* (HSNU)	China: Yunnan	PZ196775
20.	*Sphaerotheciella sinensis*	H51	*W.-Z. Huang et al., 20210529-15* (HSNU)	China: Yunnan	PZ196776

Prior to DNA extraction, visible contaminants (e.g., algae, fungi, and soil debris) were removed from all samples. Total genomic DNA was extracted using a modified CTAB protocol (Huang et al., 2019). PCR-free libraries with an insert size of 800 bp were constructed using the Illumina TruSeq™ DNA PCR-free library preparation kit (Illumina, CA, USA), and sequenced on the Illumina NovaSeq 6000 platform (Novogene, Beijing, China) to generate paired-end 150 bp reads, About 5 Gb of raw sequences were accumulated. All newly assembled plastomes were deposited in GenBank (**Table 1**).

### 2.2 Plastome assembly and annotation

Raw reads were quality-filtered by removing low-quality bases (Phred score < 30). High-quality reads were assembled using GetOrganelle v.1.7.5 (Jin et al., 2020). Initial plastome annotation was performed with CPGAVAS2 (Shi et al., 2019) and manually refined in Geneious Prime 2022 (www.geneious.com), using *Calyptothecium philippinense* Broth. (GenBank accession number: OL415138) as the reference. Genome visualization was conducted using CPGView (Liu et al., 2023).

### 2.3 Comparative analyses of plastomes

To assess structural conservation and potential rearrangements, plastomes were compared using mVISTA under the LAGAN model (Frazer et al., 2004). Expansion and contraction patterns at inverted repeat (IR) boundaries were further examined, and junction shifts among the LSC/IR/SSC regions were visualized using IRScope (https://irscope.shinyapps.io/IRplus/).

### 2.4 Phylogenetic analyses

82 protein-coding genes were aligned individually using MAFFT v.7.505 (Katoh and Standley, 2013) with the “–auto” strategy. Ambiguously aligned regions were removed manually, and the processed alignments were concatenated for downstream analyses. Phylogenetic inference was conducted using both maximum likelihood (ML) and Bayesian inference (BI). For ML analysis, IQ-TREE v2.0.6 was used (Minh et al., 2020) with the GTR+F+I+R4 substitution model, selected under the Bayesian Information Criterion (BIC) in Modeltest v.3.7 (Chernomor et al., 2016; Kalyaanamoorthy et al., 2017). Node support was evaluated with 1,000 bootstrap replicates (ML-BS). For BI analysis, MrBayes v.3.2.6 (Ronquist et al., 2012) was run under the GTR+I+G model selected by PartitionFinder2 based on the Akaike Information Criterion (AIC) (Lanfear et al., 2017). The Markov chain Monte Carlo (MCMC) algorithm was run for 5,000,000 generations, with two parallel runs and default settings, a random starting tree and tree sampling every 1000 generations. The posterior distribution of trees was summarized by a > 50% majority-rule consensus tree after discarding the first 25% of samples as burn-in. Convergence was assessed by examining likelihood plots in Tracer v.1.7 (Rambaut et al., 2018).

## 3 Result

### 3.1 Characteristics of plastomes

**Figure 1:**
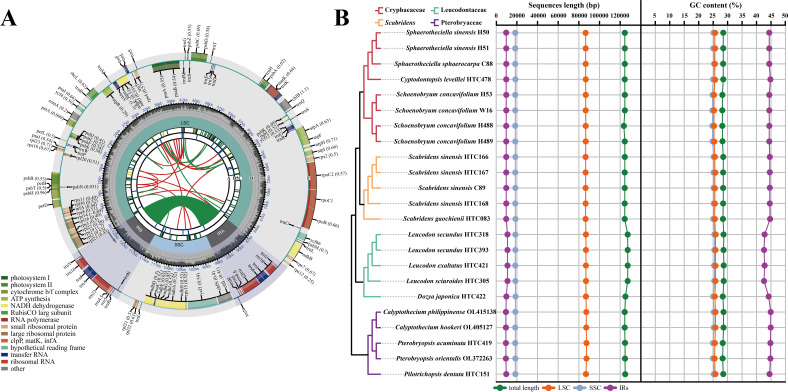
Characterization of the plastomes of Cryphaeaceae, Leucodontaceae, and Pterobryaceae species. **(A)** Representative gene map of plastomes. [The genome map includes 5 tracks. From the inside out, the first track (A) displays forward and reverse repeats connected by red and green arcs. The second track (B) shows the tandem repeats, represented by blue line segments. The third track (C) displays the microsatellite sequences, represented by green and yellow line segments. The fourth track (D) displays large single copy (LSC), small single copy (SSC), and inverted repeat (IRa and IRb). The fifth track (E) displays the GC content of the genome. The genes are distributed in the outermost circle (F), the optional codon pusage bias is displayed in parentheses after the gene name. The genes shown inside and outside of the circle are transcribed in clockwise and counterclockwise directions, respectively]. **(B)** Comparison of plastome size, and GC content in plastomes. Phylogenetic relationship within Cryphaeaceae-Leucodontaceae-Pterobryaceae clade based on CDS and maximum likelihood (ML), the sizes of the whole plastid genome, large single-copy (LSC), small single-copy (SSC), and inverted repeats (IRs) among all samples (left), along with the GC content of each region (right) are shown.

All plastomes from Cryphaeaceae, Leucodontaceae, and Pterobryaceae exhibited the typical quadripartite structure of land plant chloroplast genomes, consisting of a large single-copy (LSC) region, a small single-copy (SSC) region, and two inverted repeats (IRs) (**Figure 1A**). Total plastome size varied only slightly, ranging from 124,390 to 127,332 bp. The LSC region ranged from 86,527 to 87,405 bp, the SSC region from 18,389 to 18,706 bp, and each IR from 18,742 to 21,884 bp (**Figure 1B****;** **Supplementary Table 1**). In particular, plastomes of *Leucodon* were consistently larger (127,297 bp–127,332 bp) than those of the remaining genera (123,390 bp to 125,117 bp) (**Figure 1B****;** **Supplementary Table 1**). Overall GC content was highly conserved across taxa (28.2% to 28.7%) (**Figure 1B****;** **Supplementary Table 1**). As expected, GC content was lower in LSC (25.3%–25.8%) and SSC (24.8%–25.4%) regions than in the IR regions (42.6%–44.9%). Notably, IR GC content was lower in *Leucodon* (42.6%–42.8%) than in other genera (44.2% to 44.9%) (**Figure 1B****;** **Supplementary Table 1**).

All sampled plastomes contained 127 genes, including 82 protein-coding genes (CDS), 37 transfer RNA (tRNA) genes and 8 ribosomal (rRNA) genes (**Figure 1A****;** **Supplementary Table 2**). Eighteen genes contained introns. Among them, *rps*12, *clp*P and *ycf*3 each contained two introns, whereas *atp*F, *ndh*A, *ndh*B, *pet*B, *pet*D, *rpo*C1, *rpl*2, *rpl*16, *trn*A-UGC, *trn*G-UCC, *trn*I-GAU, *trn*K-UUU, *trn*L-UAA, *trn*V-UAC, and *ycf*66 each contained a single intron (**Supplementary Table 2**).

### 3.2 Comparative analysis of plastomes

**Figure 2:**
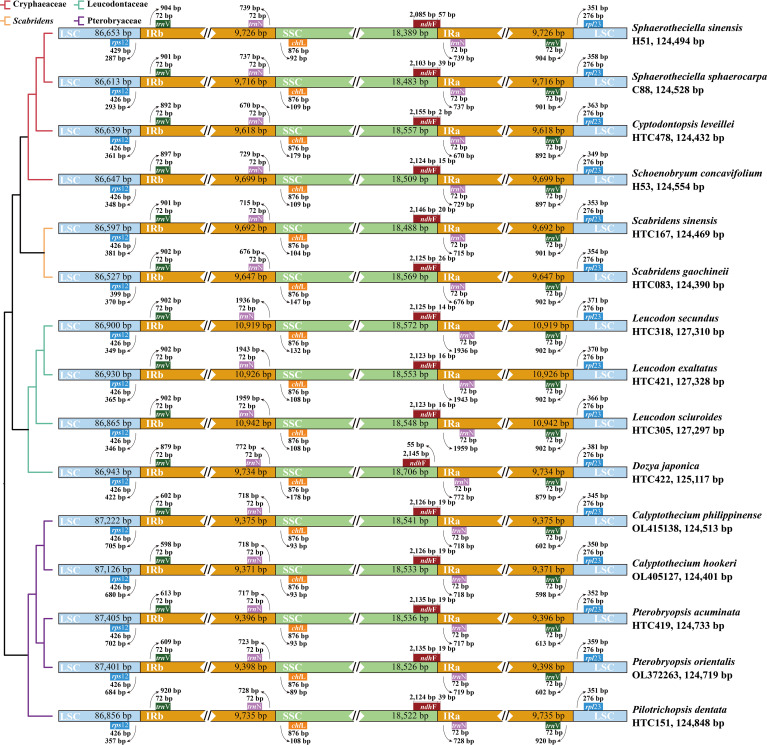
Comparisons of the large single-copy (LSC), inverted repeat a (IRa), small single-copy (SSC), and inverted repeat b (IRb) boundaries among fifteen Cryphaeaceae, Leucodontaceae, and Pterobryaceae chloroplast genomes. Phylogenetic relationship within Cryphaeaceae, Leucodontaceae, and Pterobryaceae clade based on CDS and maximum likelihood (ML) analyses.

Comparative analysis of IR boundaries revealed generally conserved plastome structure across the Cryphaeaceae, Leucodontaceae, and Pterobryaceae clades, with no significant expansion or contraction events (**Figure 2**). The *ndh*F gene spanned the SSC/IRa boundary in all taxa except *Dozya japonica*, with 2,085–2,155 bp located in the SSC and 2–57 bp in the IRa region (**Figure 2**). A lineage-specific shift was detected for *trn*N at SSC/IR junctions in *Leucodon*, where the distance to the boundary was 1,936–1,959 bp, compared with 670–772 bp in the other genera (**Figure 2**).

**Figure 3:**
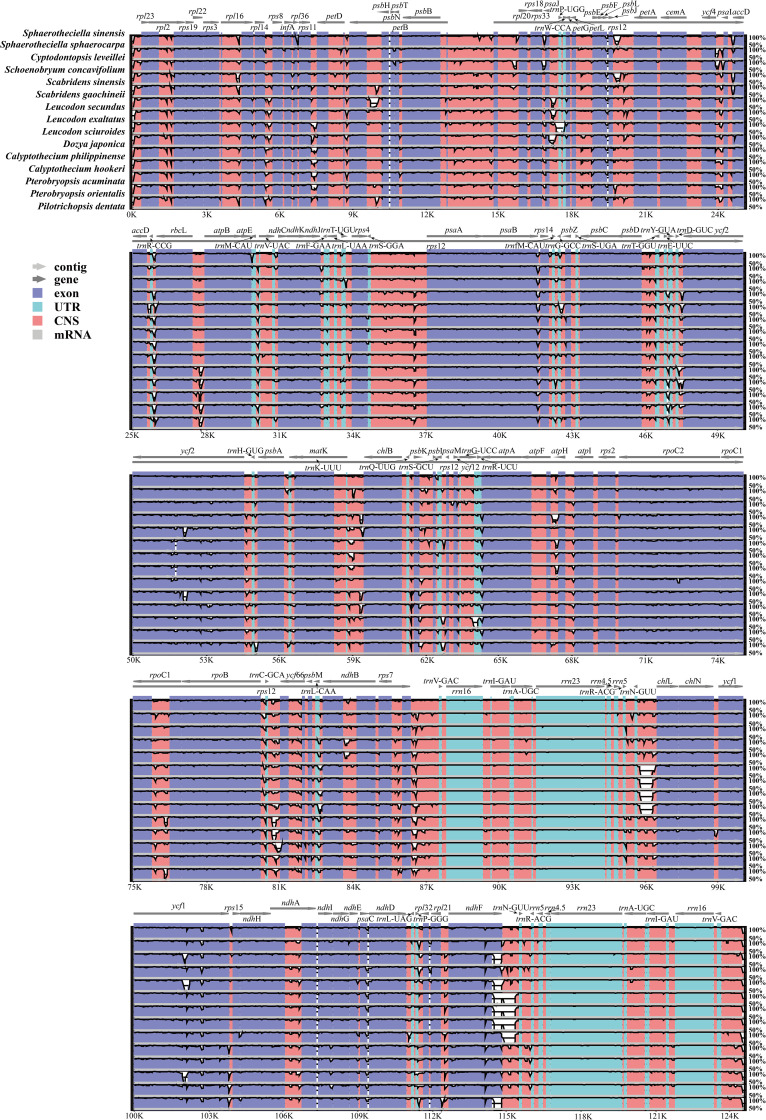
Comparative visualization of chloroplast genome sequences across fifteen Cryphaeaceae-Leucodontaceae-Pterobryaceae species. The y-axis represents sequence identity ranging from 50% to 100%, and the x-axis shows the position within the chloroplast genome. Arrows indicate the annotated genes and their transcription direction in the reference genome. The protein-coding and non-coding regions are highlighted in purple and orange, respectively.

**Figure 4:**
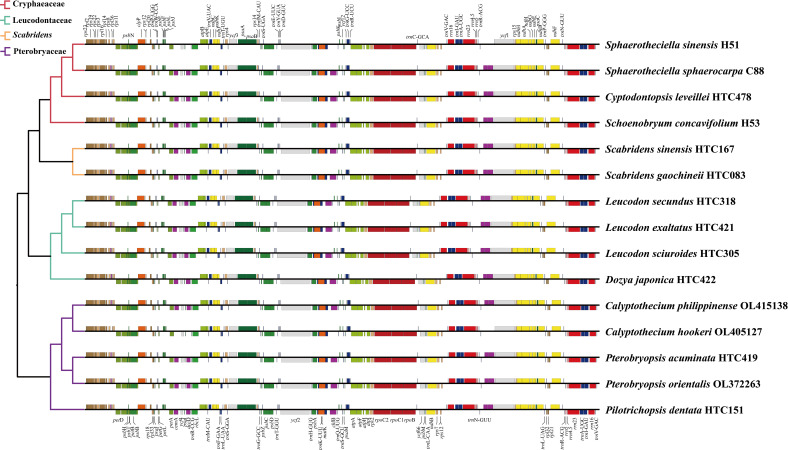
Synteny of chloroplast genomes of fifteen Cryphaeaceae, Leucodontaceae, and Pterobryaceae species. The plastomes are shown in linearized form illustrating the relative gene synteny. Names of the regions are displayed above and below the graphics.

Whole plastome comparisons showed high collinearity among the 15 representative genomes, with highly conserved gene content and order and no evidence of inversions or translocations (**Figures 3**, **4**). Sequence divergence was concentrated mainly in non-coding regions (intergenic spacers and introns), and was greater in LSC and SSC regions than in IR regions (**Figure 3**). Relatively variable regions included *rpl*12, *psb*J~*pet*A, *ycf*4~*psb*A, *rbc*L~*atp*B, *trn*M-CAU~*trn*C-UAC, *trn*S~*psb*A, *psa*B~*rps*14, *psb*D~*trn*T-GGU, *trn*D-GUC~*ycf*2, *mat*K~*chl*B, *psb*K~*psb*I, *atp*F~*atp*H, *rpo*C1, *rpo*B~*ycf*66, *ycf*66, *trn*L-CAA~*ndh*B, *ndh*A, and *rpl*21~*ndh*F (**Figure 3**).

### 3.3 Phylogenetic analyses

**Figure 5:**
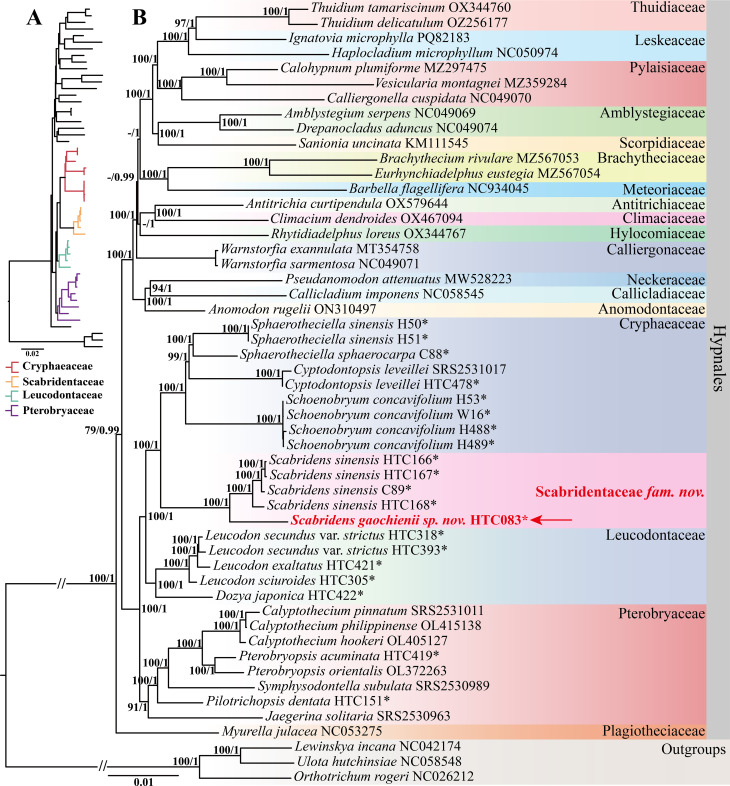
Phylogenetic tree of Hypnales inferred from concatenated 82 nucleotide sequences (analyzed using maximum likelihood (ML) **(B)** and Bayesian inference(BI) **(A)**). The phylogenetic positions of different families are highlighted in different colors. Numbers at nodes indicate ML bootstrap values (BS; before the slash) and Bayesian posterior probabilities (PP; after the slash). Dash (–) indicates nodes with ML<75 or PP<0.95. “*” means newly sequenced accessions in this study. ‘//’ means that the branch length shown in the representative picture is only half the actual length.

The concatenated dataset of 82 protein-coding genes comprised 71,517 aligned nucleotide positions, including 53,570 constant sites, 6,616 singleton sites, and 11,331 parsimony-informative sites. Maximum-likelihood (ML) and Bayesian inference (BI) analyses generated almost congruent topologies with strong support at most nodes (**Figure 5**). The ML tree, annotated with bootstrap values (BS_ML_) and Bayesian posterior probabilities (PP_BI_), is shown in **Figure 5**.

In the phylogenetic trees (**Figure 5**), all sampled Hypnales formed a maximally supported clade (PP_BI_=1, BS_ML_=100%). Within Hypnales, Cryphaeaceae, Leucodontaceae, *Scabridens*, and Pterobryaceae together formed a strongly supported lineage (PP_BI_=1, BS_ML_=100%). The unknown taxon from Motuo County, China, was sister to the four *S. sinensis* accessions (PP_BI_=1, BS_ML_=100%). Importantly, *Scabridens* was sister to Cryphaeaceae (PP_BI_=1, BS_ML_=100%) rather than to Leucodontaceae. In addition, *Dozya japonica* was sister to the *Leucodon* clade (PP_BI_=1, BS_ML_=100%), and *Pilotrichopsis dentata* (Mitt.) Besch. was nested within Pterobryaceae (PP_BI_=1, BS_ML_=91%).

## 4 Discussions

**Figure 6:**
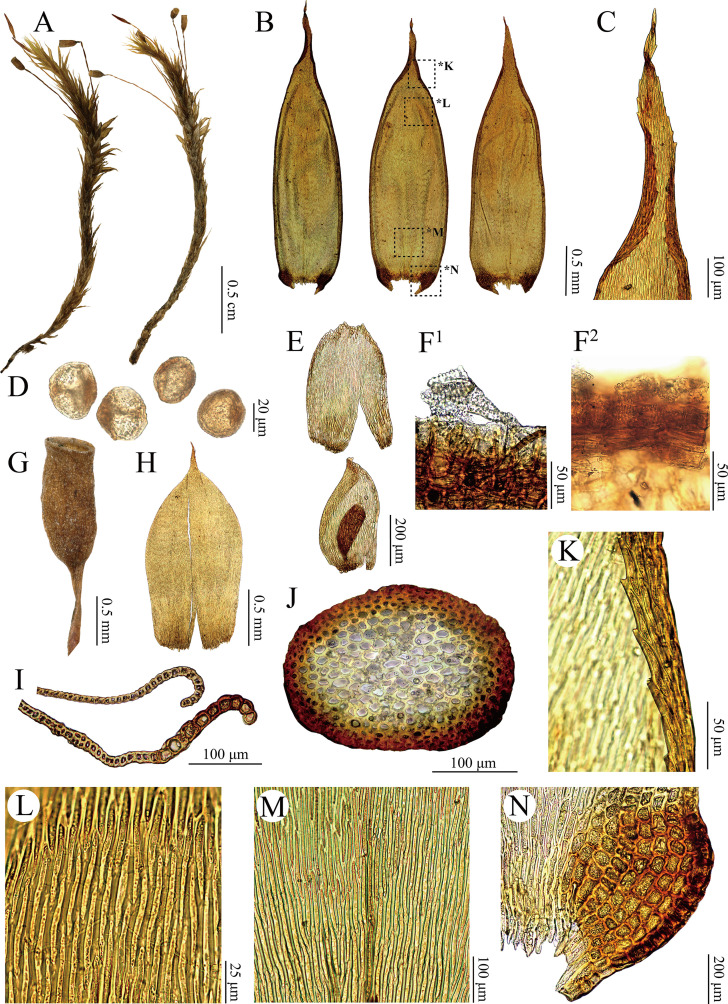
*Scabridens gaochienii* W.Z.Huang & Y.Huan Wu. **(A)** Plants; **(B)** Leaves; **(C)** Apex; **(D)** Spores; **(E)** Perigonial bracts; (F^1^) Teeth (dorsal view); (F^2^) Teeth, ventral view, show fragile and with only a residual basal portion; **(G)** Capsule; **(H)** Perichaetial leaf; **(I)** Transverse section of leaves, upper one show section in middle leaf marginal portion, and lower one show section in alar portion; **(J)** Transverse section of stem; **(K)** Upper marginal cells of leaf; **(L)** Upper laminal cells of leaf; **(M)** Basal laminal cells of leaf; **(N)** Alar cells. All from holotype specimen. Photographed by Xin-Yin Ma **(A–C, E–N)** and Yu-Si Liu **(D)**.

**Figure 7:**
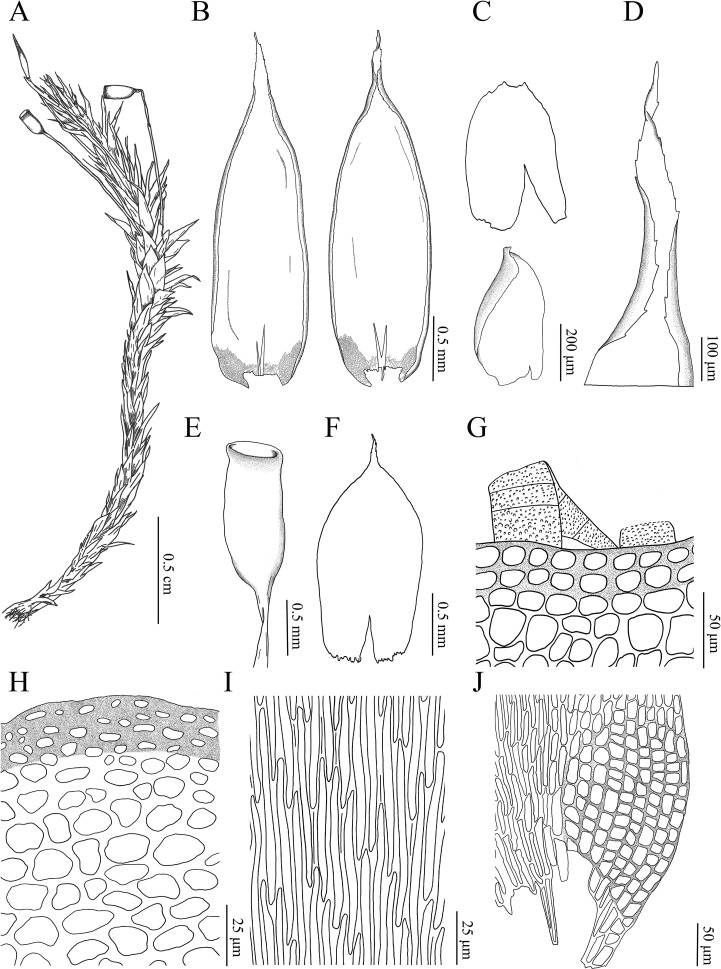
*Scabridens gaochienii* W.Z.Huang & Y.Huan Wu. **(A)** Plants; **(B)** Leaves; **(C)** Perigonial bracts; **(D)** Apex; **(E)** Capsule; **(F)** Perichaetial leavf; **(G)** Teeth; **(H)** Transverse section of stem; **(I)** Upper marginal cells of leaf; **(J)** Alar cells. All from holotype specimen. Drawn by Xin-Yin Ma.

**Figure 8:**
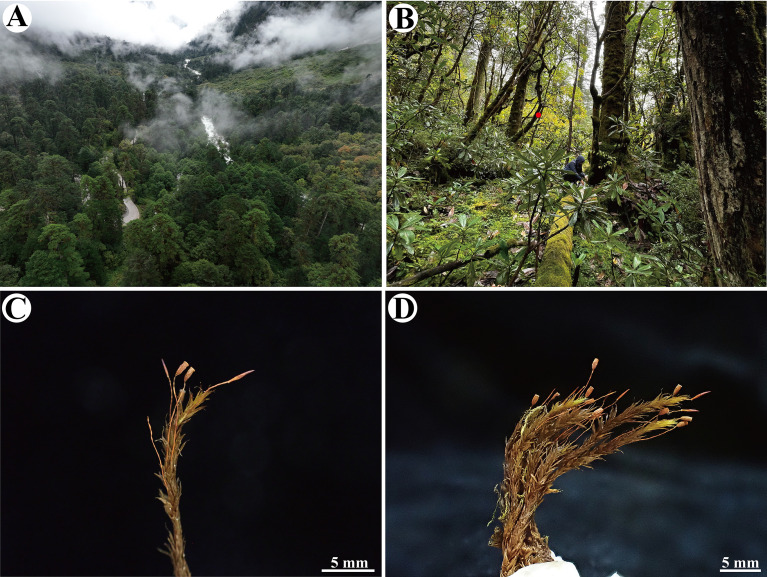
*Scabridens gaochienii* W.Z.Huang & Y.Huan Wu. **(A, B)** The habitat and locality (red dot) of the holotype specimen; **(C, D)** Plants. All from holotype specimen. Photographed by Jun Hu **(A)**, Wen-Zhuan Huang **(B)**, and Xin-Yin Ma **(C, D)**.

Our phylogenetic analyses strongly support that the unknown species from Motuo county is closely related to *Scabridens* (**Figure 5**), and morphological evidence further indicates that this species represents a distinct taxon. Compared with *S. sinensis*, this unknown taxon shows a short and often inconspicuous double costa (**Figures 6B**, **7B**), (vs. a single costa reaching the upper-middle leaf region; **Supplementary Figure 1B****;** Bartram, 1935; Zhang, 2011a, b; Zhang and He, 2011), leaf margins revolute from the base to near the apex (**Figures 6B, K**, **7B**) (vs. revolute mainly in the lower-middle part; **Supplementary Figures 1B**, **G****;** Bartram, 1935; Zhang, 2011a, b; Zhang and He, 2011), a substantially longer seta (0.55–0.70 cm; (**Figures 6A**, **7A**, **8C, D**) (vs. 0.2–0.35 cm; **Supplementary Figure 1A****;** Bartram, 1935; Zhang, 2011a, b; Zhang and He, 2011), and shorter peristome teeth (150–170 μm), usually fragile, incomplete (**Figures 6F**, **7G**) (vs. about 250 μm in length, complete; **Supplementary Figure 1I**). This species may also be confused with *Leucodon* because both share differentiated alar cells (**Figures 6B, N**, **7B, J**; Noguchi, 1989; Zhang, 2011a, b; Reese and Zander, 2014), elongate laminal cells (**Figures 6L, M**, **7I**; Noguchi, 1989; Zhang, 2011a, b; Reese and Zander, 2014), and exserted capsules (**Figures 6A**, **7A**, **8C, D**; Noguchi, 1989; Frey and Stech, 2009; Zhang, 2011a, b; Reese and Zander, 2014). However, the Motuo plants differ from *Leucodon* by having a short double costa (**Figures 6B**, **7B**), which is typically absent in *Leucodon* (Frey and Stech, 2009; Reese and Zander, 2014), an autoicous sexual condition (vs. Dioicous; Noguchi, 1989; Frey and Stech, 2009; Zhang, 2011a, b; Reese and Zander, 2014), and alar cells restricted to the basal portion (vs. alar cells extend along both sides, forming a large region; Zhang, 2011a, b; Reese and Zander, 2014).

Notably, retention of the species from Motuo County within *Scabridens* is strongly supported. First, this species matches the core diagnostic framework of *Scabridens* in overall plant architecture and reproductive morphology, including erect and simple stems, serrate upper leaf margins, and an autoicous sexual condition (Bartram, 1935; Frey and Stech, 2009). Second, molecular evidence corroborates this placement, with the Motuo lineage forming a maximally supported sister relationship to *S. sinensis*. Though this Motuo species exhibits obviously different by short double costa compared to single long costa in *S. sinensis* (Bartram, 1935; Frey and Stech, 2009; Zhang, 2011a, b), variation in costa expression is not uncommon in bryophyte genera, and transitions among single, double-short, or even reduced costae have been documented in multiple lineages (e.g., *Forsstroemia* Lindb., Frey and Stech, 2009; *Campylium* (Sull.) Spruce, Kučera and Hedenäs, 2020; *Tetrodontium* Schwägr., Ignatov and Ignatova, 2017; Lüth, 2019; and *Braunfelsia* Paris, Eddy, 1988). Therefore, recognizing the Motuo taxon within *Scabridens* is both morphologically coherent and phylogenetically robust. Together, these stable character combinations support recognition of this Motuo taxon as a species new to science, here described as *Scabridens gaochienii* W.Z.Huang & Y.Huan Wu.

The familial placement of *Scabridens* remains a central systematic issue. When this genus was first described, Bartram (1935) noted in the discussion that “the genus may not be far out of place in Leucodontaceae near *Dozya*”, because both species possess a single costa and an undifferentiated inner peristome (Noguchi, 1989; Zhang, 2011a, b). However, M. Thériot holds an opposing view, arguing that this genus may not belong to Leucodontaceae or Pterobryaceae (Bartram, 1935). Notably, a recent study by Li et al. (2024) revealed that *Scabridens sinensis* does not form a clade with *Leucodon*, but rather constitutes a sister group to species of Cryphaeaceae. Regrettably, they did not propose any formal taxonomic decisions and merely marked this species under Cryphaeaceae in their appendix phylogeny tree (Li et al., 2024). Similarly, our phylogenetic results also show that *Scabridens* and Cryphaeaceae form a sister group (**Figure 5**). Whether *Scabridens* should be classified within Cryphaeaceae?

According to the morphological definition of Cryphaeaceae in the protologue (Schimper, 1856) and various monograph (e.g., Noguchi, 1989; Frey and Stech, 2009; Zhang, 2011a; Reese and Zander, 2014), this family was characterized by: (1) Costa single, percurrent to excurrent; (2) seta very short, capsule typically immersed; (3) lamina cells short, smooth or prorate; and (4) perichaetial leaves often with long-aristate. On the contrary, *Scabridens* exhibits (1) a short double costa or a single costa (**Figure 6B**, **7B****;** **Supplementary Figure 1B****;** Bartram, 1935; Frey and Stech, 2009; Zhang, 2011a, b), (2) long setae with exserted capsules (**Figures 8C, D**, **6A**, **7A****;** **Supplementary Figure 1A**; Bartram, 1935; Frey and Stech, 2009; Zhang, 2011a, b), (3) rhomboid to elongate laminal cells (**Figures 6L, M**, **7I****;** **Supplementary Figures 1G, K, L, M**; Bartram, 1935; Zhang, 2011a, b), and mucronate perichaetial leaves (**Figures 6H**, **7F****;** **Supplementary Figure 1H**). These significant morphological differences (**Table 2**) do not support the placement of *Scabridens* within Cryphaeaceae, unless we expand the definition of Cryphaeaceae. However, extending Cryphaeaceae to encompass these differences would substantially blur family-level morphological boundaries among Cryphaeaceae, Leucodontaceae, and Pterobryaceae, and would create additional taxonomic problems (Frey and Stech, 2009). Therefore, we propose to elevate *Scabridens* to family rank as Scabridentaceae W.Z.Huang & Y.Huan Wu.

**Table 2:** Difference among Cryphaeaceae, Leucodontaceae, and Scabridentaceae.

Family	Cryphaeaceae	Leucodontaceae	Scabridentaceae
Plants	Medium-sized, stems erect to pendulous, often sparsely, regularly or irregulary pinnately branched	Medium-sized to large, stems simple or sparsely branched to feondose	Medium-sized, stems erect or sparingly branched
Central strand	Absent	Weak or absent	Absent
Leaf	Ovate to narrowly lanceolate	Broadly ovate to lanceolate	Broadly ovate at base and gradually narrowed to an attenuate apex
Sexual condition	Autoicous, rarely dioicous	Dioicous	Autoicous
Costa	Single, percurrent to excurrent.	Single, reach the middle-upper part of the leaf, or absent	Single, reach the middle-upper part of the leaf, or short double
Laminal cells	Short, smooth or prorate	Oval to elongate, smooth to rarely prorulate	Rhomboid to elongate, smooth
Alar cells	Differentiated, homochromatic	Differentiated, homochromatic	Differentiated, reddish brown to brownish black
Perichaetial leaf	With long-aristate	With mucronate apex.	With mucronate apex.
Setae	Very short, capsule immersed	Rather short to elongate, capsule usually exserted	Elongate, capsules exserted
Teeth	Exostome teeth papillose, endostome ± reduced, rarely absent	Exostome teeth papillose; endostome susally rudimentary	Peristome teeth papillose, endostome reduced

Additionally, our results also support a formal transfer of *Pilotrichopsis* Besch. to Pterobryaceae. Altuough *Pilotrichopsis* has traditionally been placed in Cryphaeaceae (e.g., Brotherus, 1905; Buck, 1998; Buck et al., 2000; Frey and Stech, 2009; Jia and He, 2025; Sugimura and Uzawa, 2025), molecular studies have repeatedly shown that *Pilotrichopsis dentata* is nested within Pterobryaceae (Cox et al., 2010; Li et al., 2024), a pattern corroborated here (**Figure 5**). Over recent decades, molecular phylogenetics has substantially reshaped the traditional classification of bryophytes (Bechteler et al., 2023; Li et al., 2024; Xu et al., 2026), given that monophyly is a primary criterion for modern family delimitation, retaining *Pilotrichopsis* in Cryphaeaceae is no longer tenable. We therefore adopt the taxonomic transfer of *Pilotrichopsis* to Pterobryaceae.

## 5 Taxonomy

Scabridentaceae W.Z.Huang & Y.Huan Wu, fam. nov.

*Diagnosis:* *—Plants medium-sized, erect or sparingly branched. Stem cross-section without a central strand. Leaves densely clustered, slightly concave, not plicate, broadly ovate at the base and gradually narrowed to an attenuate apex. Margins revolute from the base to near the apex or only in the lower half, serrate in the upper portion, crenulate or nearly smooth in the middle and lower portion. Costa single, reach the middle-upper part of the leaf, or double and short. Laminal cells rhomboid to elongate, alar cells well differentiated, quadrate to short rectangular. Sexual condition autoicous. Perichaetial leaves are smaller than stem leaves, ecostate. Setae long, capsules exserted. Stomata superficial at urn base. Peristome teeth 16, endostome reduced, papillose throughout.*

Type Genus:—*Scabridens* E.B.Bartram, Ann. Bryol. 8: 16. 1935.

Included species: *Scabridens sinensis* E.B.Bartram, and *S. gaochienii* W.Z.Huang & Y.Huan Wu.

Emended diagnosis: Consistent with the diagnosis of Scabridentaceae.

Type species: *Scabridens sinensis* E.B.Bartram, Ann. Bryol. 8: 16. 1935.

Type: China. Kweichow (Guizhou) Province, Nin Tao Shan, on bark, alt. 2000 m., 2 October 1931, *S. Y. Cheo 825a* (holotype: FH [00220367], n. v.; isotypes: FH [00220368], n.v.; NY [00845582], n. v.; PC [101249], n. v.).

Illustrations and descriptions: Bartram (1935): 16–17, Figure 10), Chen (1978): 35–37, Figure 214), Zhang (2005): 86, Figure 33), Zhang (2011a: 189–190, Figure 81), Zhang and He (2011): 180–181, Figure 323), Zhang (2011b: 347–348, Figure 193), Zhang et al. (2016): 273–274, Figure 34–37), and **Supplementary Figure 1**.

*Scabridens gaochienii* W.Z.Huang & Y.Huan Wu, sp. *nov.* (**Figures 6**–**8**).

Chinese name: 高氏疣齿藓 [Gāo Shì Yóu Chǐ Xiǎn].

Diagnosis: *Similar to Scabridens sinensis, but can be distinguished by the short double costa, revolute margins from the base to near the apex, longer seta (0.55–0.70 cm), and shorter peristome teeth (150–170 μm), usually fragile and with only a residual basal portion on oral.*

Type: China. Xizang Autonomous Region, Linzhi City, Motuo County, Yarlung Zangbu Grand Canyon National Nature Reserve, from Pai Town to Beibeng Village, along Paimo Road, Xiaoyandong, 29°24′56.52″ N, 95°4′7.88″ E, 2691 m a.l.t., on tree trunk,12 Oct. 2024, *W.-Z. Huang & F.-Y. Zhang 20241012-71* (holotype: HTC!; isotype: HSNU!).

Description: Plants in loose tufts, yellowish green in upper part and dark brown below. Stems erect, 2.3–3.5 cm high, simple or sparingly branched, with brownish to dark rhizoids at base. Cross-section of stem rounded to oval, epidermis with 2–4(–5) layers of smaller, brownish, thick-walled cells, and 9–11 rows of larger lumen, thick-walled inner cortical cells, central strand absent. Leaves densely clustered, slightly concave, without plicate, appressed or erecto-patent when dry, erect spreading when moist, broadly ovate at base and gradually narrowed into an attenuate apex, broadest slightly above the middle, apex usually twisted, 2.7–3.3 × 0.65–0.8 cm. Margins revolute from the base to near the apex, serrate in the upper portion, crenulate or nearly smooth in the middle and lower portion. Costa slender, double, short, usually not obvious. Upper and middle laminal cells rhomboid or linear, 55–73 × 3.5–5.5 μm; basal laminal cells linear, 70–95 × 8–12 μm; alar cells well differentiated, reddish brown to dark brown, quadrate or short rectangular, 33–65 × 25–42 μm, unistratose. Sexual condition autoicous. Androecium small, axillary, perigonial bract concave, tongue-shaped or broadly ovate and short acuminate, costa absent, ca. 0.5 mm long, 0.3 mm wide. Perichaetial leaves are smaller than stem leaves, ovate-lanceolate, broadest at the middle, serrate near apex, mucronate, without a costa. Setae yellowish-brown, smooth, 0.55–0.70 cm long, helically coiled near capsules. Capsules yellowish-brown, oblong-cylindric, 1.2–1.4 × 0.55–0.68 mm; exothecial cells irregular hexagonal, thick-walled; stomata superficial, only present at the urn base. Annulus not seen. Peristome teeth 16, exostome lanceolate, 150–170 μm in length, pellucid, papillose, usually fragile and with only a residual basal portion on oral, endostome undeveloped. Spores 28–40 μm in diameter, papillose under the light microscope.

**Etymology.** The species epithet is to honor an early Chinese bryologist, Qian Gao, in appreciation for his important contributions to our knowledge of the Chinese bryoflora.

**Figure 9:**
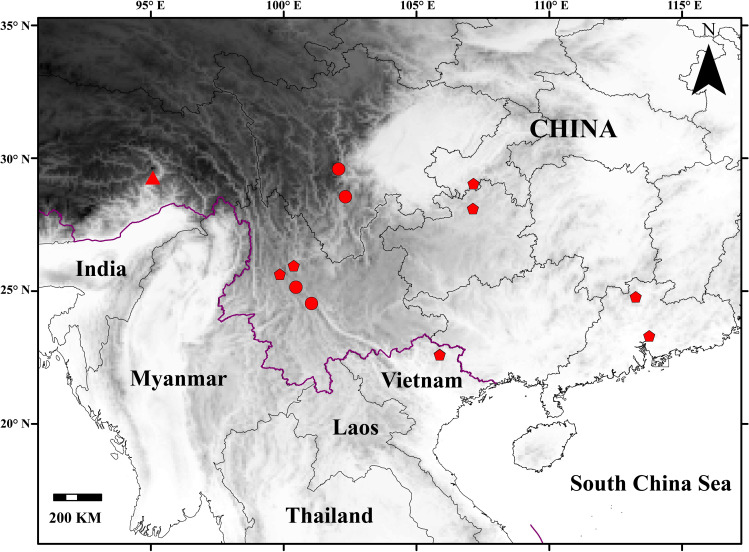
Distribution of *Scabridens gaochienii* and *S. sinensis*. Red triangles: *S. gaochienii* collection sites; red pentagons: *S. sinensis* historical records; red circles: *S. sinensis* newly discovered sites in this study.

**Distribution and habitat.** *Scabridens gaochienii* is currently known only from the Yarlung Zangbu Grand Canyon National Nature Reserve in Motuo County, Linzhi City, Xizang Autonomous Region, China (**Figure 9**). The species grows on the tree trunk in an alpine coniferous forest at an elevation of 2,691 meters. The forest mainly comprises *Abies delavayi* var. *motuoensis* W.C.Cheng & L.K.Fu, *Ilex fragilis* Hook. f., *Tsuga dumosa* (D.Don) Eichler, *Sorbus thibetica* (Card.) Hand.-Mazz., etc (**Figures 8A, B**). In our collected, *S*. *gaochienii* is found only in association with *Symblepharis vaginata* (Hook. ex Harv.) Wijk & Margad.

## 6 Key to the species of *Scabridens*

1. Costa single, reach middle-upper part of leaf; margins revolute from base to middle; seta short, 0.2–0.3 cm; peristome teeth ca. 250 μm, complete…*Scabridens sinensis*.

1. Costa short, double; margins revolute from base to near apex; seta 0.55–0.70 cm; peristome teeth 150–170 μm, usually fragile, incomplete…*Scabridens gaochienii*.

## Data Availability

The data presented in the study are deposited in the NCBI (https://www.ncbi.nlm.nih.gov/) repository. The names of the repository/repositories and accession number(s) can be found in the article/**Supplementary Material**.
