# Supplementary material for: New moss taxa from Eastern Himalaya: Plastid genomics supports the establishment of Scabridentaceae fam. nov. and the description of Scabridens gaochienii sp. nov. (Hypnales, Bryophyta)
Source: Front Plant Sci. 2026 Jun 9;17:1842480. doi: 10.3389/fpls.2026.1842480 (PMC13287142; doi:10.3389/fpls.2026.1842480)
Supplement: Supplementary file 1 [file Table1.docx]

***Supplementary Material***

**Supplementary Figures and Tables**

**Table S1** Complete features of 23 Cryphaeaceae-Leucodontaceae-Pterobryaceae plastid genomes plastomes**.**

| **Species** | **DNA Code** | **Genome size (bp)** | **LSC Length (bp)** | **SSC Length (bp)** | **IR Length (bp)(×2)** | **Total GC content (%)** | **LSC GC content (%)** | **SSC GC content (%)** | **IR GC content (%)** |
| --- | --- | --- | --- | --- | --- | --- | --- | --- | --- |
| *Calyptothecium philippinense* | OL415138 | 124513 | 87222 | 18541 | 9375 | 28.6 | 25.8 | 25.3 | 44.9 |
| *Calyptothecium hookeri* | OL405127 | 124401 | 87126 | 18533 | 9371 | 28.6 | 25.8 | 25.3 | 44.9 |
| *Cyptodontopsis leveillei* | HTC478 | 124432 | 86639 | 18557 | 9618 | 28.5 | 25.6 | 25.0 | 44.8 |
| *Dozya japonica* | HTC422 | 125117 | 86943 | 18706 | 9734 | 28.4 | 25.6 | 25.0 | 44.2 |
| *Leucodon exaltatus* | HTC421 | 127328 | 86923 | 18553 | 10926 | 28.7 | 25.8 | 25.4 | 42.8 |
| *Leucodon sciuroides* | HTC305 | 127297 | 86865 | 18548 | 10942 | 28.7 | 25.8 | 25.5 | 42.6 |
| *Leucodon secundus* var. *strictus* | HTC318 | 127310 | 86900 | 18572 | 10919 | 28.7 | 25.8 | 25.5 | 42.8 |
| *Leucodon secundus* var. *strictus* | HTC393 | 127332 | 86925 | 18545 | 10931 | 28.6 | 25.8 | 25.5 | 42.6 |
| *Pilotrichopsis dentata* | HTC151 | 124848 | 86856 | 18522 | 9735 | 28.5 | 25.7 | 25.3 | 44.5 |
| *Pterobryopsis acuminata* | HTC419 | 124733 | 87405 | 18536 | 9396 | 28.4 | 25.6 | 25.0 | 44.8 |
| *Pterobryopsis orientalis* | OL372263 | 124719 | 87401 | 18530 | 9394 | 28.3 | 25.5 | 25.0 | 44.8 |
| *Scabridens sinensis* | HTC166 | 124498 | 86613 | 18487 | 9699 | 28.5 | 25.6 | 25.2 | 44.6 |
| *Scabridens sinensis* | HTC167 | 124469 | 86597 | 18488 | 9692 | 28.5 | 25.6 | 25.2 | 44.6 |
| *Scabridens sinensis* | C89 | 124483 | 86611 | 18444 | 9692 | 28.5 | 25.6 | 25.2 | 44.6 |
| *Scabridens sinensis* | HTC168 | 124453 | 86542 | 18487 | 9712 | 28.5 | 25.6 | 25.2 | 44.6 |
| *Scabridens gaochienii* | HTC083 | 124390 | 86527 | 18569 | 9647 | 28.4 | 25.5 | 25.1 | 44.7 |
| *Schoenobryum concavifolium* | H53 | 124554 | 86647 | 18509 | 9699 | 28.2 | 25.3 | 24.8 | 44.4 |
| *Schoenobryum concavifolium* | HTC488 | 124586 | 86679 | 18511 | 9698 | 28.2 | 25.3 | 24.8 | 44.4 |
| *Schoenobryum concavifolium* | HTC489 | 124548 | 86640 | 18510 | 9699 | 28.2 | 25.3 | 24.8 | 44.4 |
| *Schoenobryum concavifolium* | W16 | 124554 | 86646 | 18510 | 9699 | 28.2 | 25.3 | 24.8 | 44.4 |
| *Sphaerotheciella sphaerocarpa* | C88 | 124528 | 86613 | 18483 | 9716 | 28.5 | 25.6 | 25.4 | 44.4 |
| *Sphaerotheciella sinensis* | H50 | 124494 | 86653 | 18389 | 9726 | 28.5 | 25.6 | 25.1 | 44.4 |
| *Sphaerotheciella sinensis* | H51 | 124494 | 86653 | 18389 | 9726 | 28.5 | 25.6 | 25.1 | 44.4 |

**Table** **S2** Genes contents in the plastomes of Cryphaeaceae-Leucodontaceae-Pterobryaceae species*.*

| **Category** | **Groups** | **Name** |
| --- | --- | --- |
| Photosynthesis-related genes | Rubisco | *rbcL* |
|  | Photosystem I | *psaA*, *psaB*, *psaC*, *psaI*, *psaJ*, *psaM* |
|  | Photosystem II | *psbA*, *psbB*, *psbC*, *psbD*, *psbE*, *psbF*, *psbH*, *psbI*, *psbJ*, *psbK*, *psbL*, *psbM, psbN*, *psbT*, *psbZ* |
|  | ATP Synthase | *atpA*, *atpB*, *atpE*, *atpF*^a*^, *atpH*, *atpI* |
|  | Cytochrome b/f complex | *petA*, *petB*^a*^, *petD*^a*^, *petG*, *petL* |
|  | NADPH dehydrogenase | *ndhA*^a*^, *ndhB*^a*^, *ndhC*, *ndhD*, *ndhE*, *ndhF*, *ndhG*, *ndhH*, *ndhI*, *ndhJ*, *ndhK* |
|  | Chlorophyll biosynthesis | *chlB*, *chlL*, *chlN* |
| Transcription and translation related genes | Transcription | *rpoB*, *rpoC1*^a*^*, rpoC2* |
|  | Ribosomal proteins | *rps2*, *rps3*, *rps4*, *rps7*, *rps8*, *rps11*, *rps12*^a*^, *rps14*, *rps15*, *rps18*, *rps19*, *rpl2* ^a*^*, rpl14*, *rpl16* ^a*^, *rpl20*, *rpl21*, *rpl22*, *rpl23*, *rpl32*, *rpl33*, *rpl36* |
|  | Translation initiation factor | *infA* |
| RNA genes | Ribosomal RNA | *rrn5*^b^(×2), *rrn4.5*^b^(×2), *rrn16*^b^(×2), *rrn23*^b^(×2) |
|  | Transfer RNA | *trnA-UGC*^a*,b^(×2)*, trnC-GCA*, *trnD-GUC*, *trnE-UUC, trnF-GAA*, *trnfM-CAU*, *trnG-UCC*^a*^, *trnH-GUG*, *trnI-GAU*^a*,b^ (×2), *trnK-UUU*^a*^, *trnL-UAG*, *trnL-CAA*, *trnL-UAA*^a*^, *trnM-CAU*(×2)*, trnN-GUU*^b^(×2), *trnP-GGG*, *trnP-UGG, trnQ-UUG*, *trnR-ACG*^b^(×2)*, trnR-CCG*, *trnR-UCU*, *trnS-GCU*, *trnS-GGA*, *trnS-UGA*, *trnT-GGU*, *trnT-UGU*, *trnV-GAC*^b^(×2), *trnV-UAC*^a*^, *trnW-CCA*, *trnY-GUA* |
| Other genes | RNA processing | *matK* |
|  | Carbon metabolism | *cemA* |
|  | Fatty acid synthesis | *accD* |
|  | Proteolysis | *clpP^a**^* |
| Genes of unknown function | Conserved reading frame | *ycf1, ycf3^a**^, ycf4*, *ycf12, ycf66^a*^* |

a, genes containing introns; superscript *, the number of introns in the gene; b, genes located in IR regions, ×2, genes with two copies.


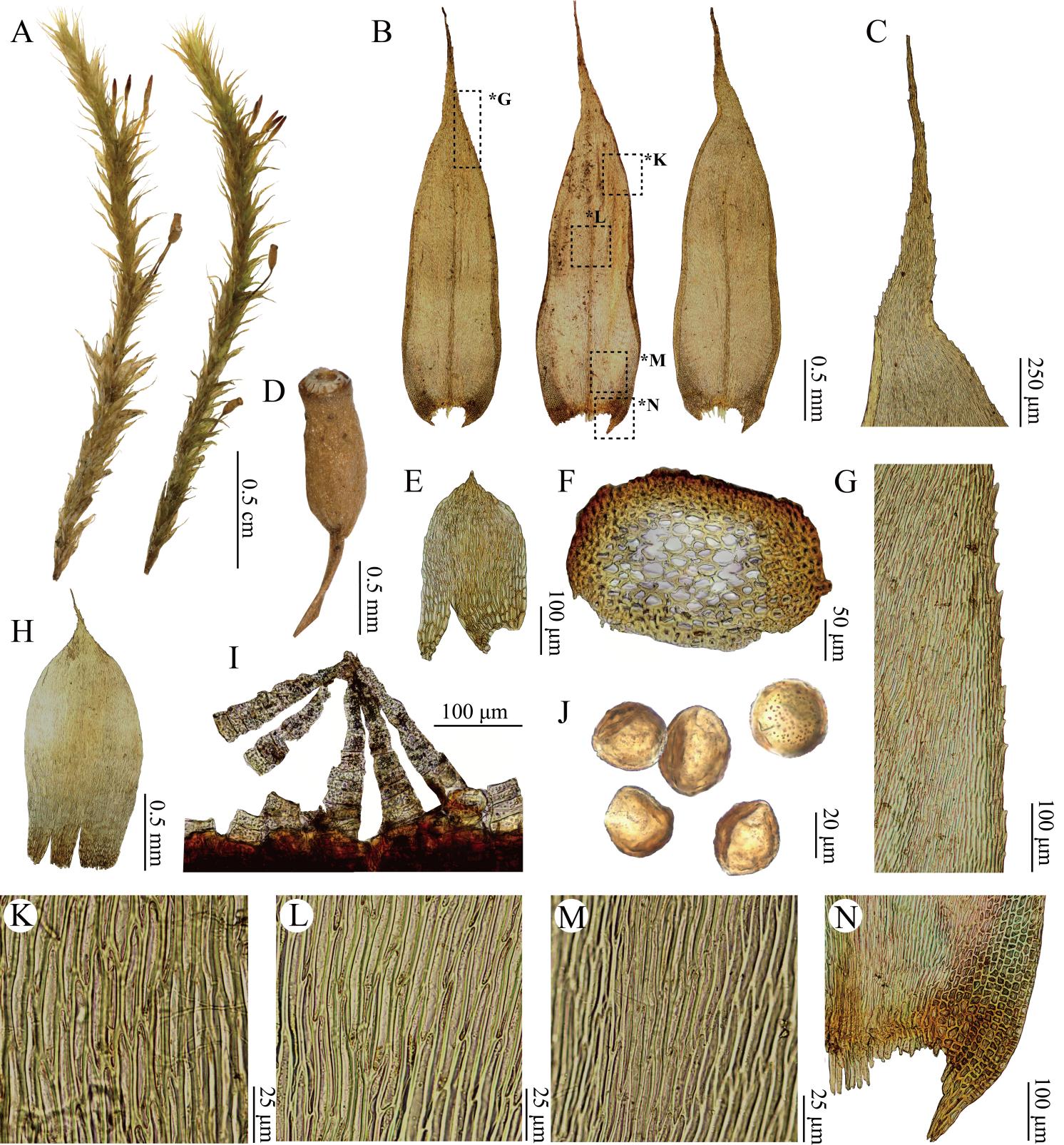


**Supplementary Figure 1.** *Scabridens sinensis* E.B.Bartram. (A) Plants; (B) Leaves; (C) Apex; (D) Capsule; (E) Perigonial bracts; (F) Cross-section of stem; (G) Upper marginal cells of leaf; (H) Perichaetial leaf; (I) Teeth; (J) Spores; (K) Upper laminal cells of leaf; (L) Middle laminal cells of leaf; (M) Basal laminal cells of leaf; (N) Alar cells of leaf. All from *W.-Z. Ma 20-10936* (KUN). Photographed by Xin-Yin Ma (A–I, K–N) and Yu- Si Liu (J).


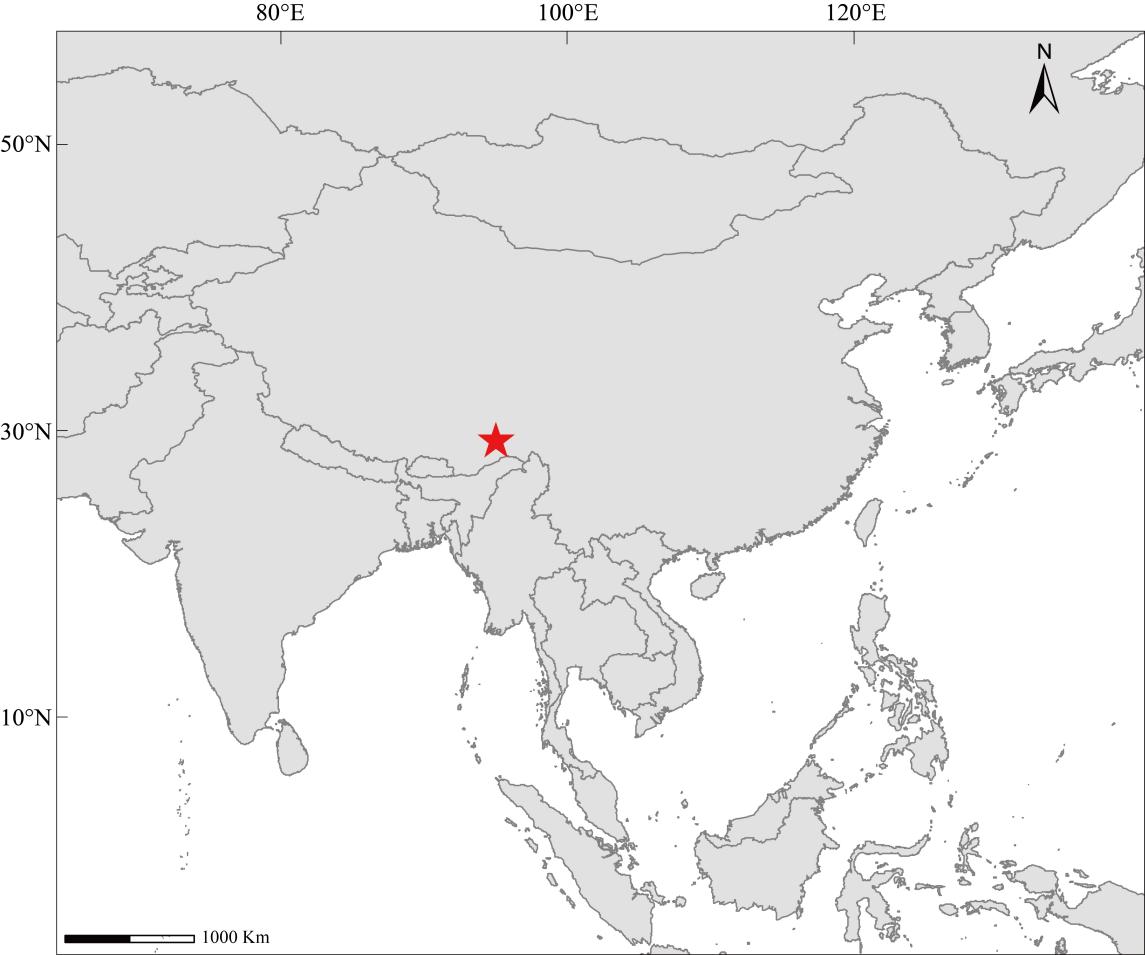


**Supplementary Figure 2.** The location of the Yarlung Zangbu Grand Canyon National Nature Reserve in Motuo County, Linzhi City, Xizang Autonomous Region, China.
